# A Mini Library of Novel Triazolothiadiazepinylindole Analogues: Synthesis, Antioxidant and Antimicrobial Evaluations

**DOI:** 10.1155/2014/581737

**Published:** 2014-02-20

**Authors:** Jaiprakash Sharanappa Biradar, Parveen Rajesab, Naveen Jaiprakash Biradar, Sasidhar Balappa Somappa

**Affiliations:** ^1^Central Research Lab, Department of Chemistry, Gulbarga University, Gulbarga, Karnataka State 585 106, India; ^2^Smt. V.G. Degree College for Women, Gulbarga, Karnataka State 585 102, India; ^3^Organic Chemistry Section, Chemical Science and Technology Division, National Institute for Interdisciplinary Science and Technology (CSIR), Thiruvanthapuram-695 019, Kerala, India

## Abstract

A new series of novel triazolothiadiazepinylindole analogues were synthesized with an aim to examine possible antioxidant and antimicrobial activities. The titled compounds (**3a–z**) were obtained in good yield by reacting 5-(5-substituted-3-phenyl-1H-indol-2-yl)-4-amino-4H-1,2,4-triazole-3-thiols **1a–c** with 3-(2,5-disubstituted-1H-indol-3-yl)-1(4-substituted phenyl)prop-2-en-1-ones **2a–i**. All the newly synthesized compounds were characterized by IR, ^1^H NMR, mass spectroscopic and analytical data. The synthesized analogues were tested for antioxidant and antimicrobial potency. Among the tested compounds **3a–c** and **3j–l** have shown very promising free radical scavenging activity and total antioxidant capacity. Compounds **3d–f**, **3m–o**, and **3s–z** have shown excellent ferric reducing antioxidant activity. An outstanding antimicrobial activity is observed with compounds **3a–c** and **3j–l**.

## 1. Introduction

Antioxidants [1–3] act as “free radical scavengers” hence to prevent or slow the damage done by the free radicals [4–6]. Free-radical-induced oxidative stress associated with several cellular toxic processes including oxidative damage to protein, and DNA, membrane lipid oxidation, enzyme inactivation, and gene mutation leads to carcinogenesis [7]. Antioxidants are involved in processes such as immunity, protection against tissue damage, and reproduction and prevent growth or development caused by free radicals [8–10]. Antioxidants are useful in the prevention and treatment of Parkinson's and Alzheimer's disease [11–13].

Heterocycles constitute one of the major areas of organic chemistry and play important roles in drug discovery. Many of the best selling drugs currently in use contain one or more heterocyclic rings. Several fused heterocycles as well as biheterocycles are referred to as privileged structures [14]. Among them, sulfur- and nitrogen-containing heterocyclic compounds have maintained the interest of researchers and their unique structures led to several applications in different areas [15]. Triazoles and their derivatives constitute an important class of heterocyclic compounds and their analogues have been reported to possess various biological activities such as antimicrobial [16], anti-inflammatory [17], antihypertensive, anti-HIV [18], anticancer, and antitumor [19, 20]. Several compounds containing 1,2,4-triazole rings known as drugs like fluconazole, posaconazole, alprazolam, [21] and triazolothiadiazepine analogues represent a well-known class of drug substances at different stages of research, which possess antiviral [22] and antimicrobial properties [23].

Indole is a heterocycle of great importance in biological systems [24, 25]. The indole moiety is present in a number of drugs currently [26] in the market; in our previous approaches, we have described some new indole analogues with highly potent antioxidant, DNA cleavage and antimicrobial activities [27–30].

Interestingly, we have developed a new green protocol for the synthesis of rapid and clean synthetic route towards mini library of triazolothiadiazepinylindole analogues, which showed *in vitro* antioxidant and antimicrobial activities.

## 2. Materials and Methods

### 2.1. Chemistry

All chemicals used in this investigation were of analytical grade and were purified whenever necessary. Melting points of the synthesized compounds were measured in open capillaries and are uncorrected. Reactions were monitored by thin-layer chromatography (TLC) on silica gel 60 F_254_ aluminium sheets (MERCK). Iodine vapour was used as detecting agent. IR spectra were recorded in KBr on PerkinElmer and FTIR spectrophotometer (*ν*_max⁡_ in cm^−1^). ^1^H NMR and ^13^C NMR spectra on BRUKER AVENCE II 400-MHz NMR spectrometer and the chemical shifts were expressed in ppm (*δ* scale) downfield from TMS as an internal reference. The mass spectra were recorded on LC-MSD-Trap-SL instrument. The elemental analysis was performed by using FLASH EA 1112 SERIES instrument.

#### 2.1.1. General Procedure for the Synthesis of Compounds **1a**–**c**

The precursors 5-(5-substituted-3-phenyl-1H-indol-2-yl)-4-amino-4H-1,2,4-triazole-3-thiols) (**1a**–**c**) were obtained from 3,5-disubstituted indol-2-carboxyhydrazides by reported method [31].

#### 2.1.2. General Procedure for the Synthesis of Compounds **2a**–**i**

3-(2,5-disubstituted-1H-indol-3-yl)-1(4-substituted phenyl) prop-2-en-1-one **2a–i **were prepared by reported method [29] by reacting disubstituted indole aldehydes with substituted acetophenone in the presence of piperidine in good yields.

#### 2.1.3. General Procedure for the Synthesis of Compounds **3a**–**z**

*(1) Conventional Method.* To a solution of substituted indolyl- triazole **1a**–**c** (0.01 mol) in acetic acid substituted chalcones **2a**–**i** (0.01 mol) were added. The reaction mixture was refluxed 3-4 hrs. The completion of the reaction was monitored by TLC. After the completion, the reaction mixture was poured to a beaker containing 100 mL of ice-cold water. The crude products thus separated were filtered and recrystallized from ethanol to yield target compounds **3a**–**z**.

*(2) Microwave Oven Method.* A mixture of substituted indolyl triazole **1a**–**c** (0.01 mol) and substituted chalcones **2a**–**i** (0.01 mol) was powdered, mixed, and introduced to borosil sample crucible containing few drops of acetic acid. This was subjected to microwave irradiation for 10 minutes with 70% microwave power. After the completion (TLC), reaction mixture was brought to room temperature, washed with ethanol, and recrystallized to get the title compounds **3a**–**z** which were found to be in good purity (TLC) and excellent yield.

*8-(5-Chloro-2-phenyl-1H-indol-3-yl)-3-(5-chloro-3-phenyl-1H-indol-2-yl)-6-(4-chlorophenyl)-[1,2,4]triazolo[3,4-b][1,3,4]thiadiazepine (**3a**)*. IR (KBr) *ν*_max⁡_ (cm^−1^): 3180, 3090, 1654, 1624, 1546; ^1^H NMR (DMSO-d_6_+ CDCl_3_) *δ* (ppm): 12.47 (s, 1H, indole NH), 11.63 (s, 1H, indole NH), 7.31–8.23 (m, 20H, Ar-H), 5.65 (s, 1H, –CH=); ^13^C NMR (DMSO-d_6_+ CDCl_3_) *δ* (ppm): 108, 111, 113, 117, 118, 118, 118, 120, 123, 125, 126, 126, 126, 128, 128, 128, 128, 129, 129, 129, 130, 132, 133, 134, 135, 138, 138, 144, 145, 166. MS: *m/z* = 712 [M]^+∙^, 714 [M+2], 718 [M+4], 720 [M+6]; Anal. calcd. for (C_39_H_23_N_6_Cl_3_S): C, 65.60; H, 3.25; N, 11.77%. Found: C, 65.59; H, 3.21; N, 11.75%.

*8-(5-Chloro-2-phenyl-1H-indol-3-yl)-3-(5-chloro-3-phenyl-1H-indol-2-yl)-6-phenyl-[1,2,4]triazolo[3,4-b][1,3,4]thiadiazepine (**3b**)*. IR (KBr) *ν*_max⁡_ (cm^−1^): 3189, 3049, 1608, 1579, 1553; ^1^H NMR (DMSO-d_6_+ CDCl_3_) *δ* (ppm): 11.15 (s, 1H, indole NH), 10.25 (s, 1H, indole NH), 7.29–8.72 (m, 21H, Ar-H), 4.95 (s, 1H, –CH=); MS: *m/z* = 678 [M]+∙, 680 [M+2], 682 [M+4]; Anal. calcd. for (C_39_H_24_N_6_Cl_2_S): C, 68.92; H, 3.56; N, 12.37%. Found: C, 68.81; H, 3.52; N, 12.31%.

*8-(5-Chloro-2-phenyl-1H-indol-3-yl)-3-(5-chloro-3-phenyl-1H-indol-2-yl)-6-(4-methylphenyl[1,2,4]triazolo[3,4-b][1,3,4]thiadiazepine (**3c**).* IR (KBr) *ν*_max⁡_ (cm^−1^): 3108, 3053, 1606, 1574, 1553; ^1^H NMR (DMSO-d_6_+ CDCl_3_) *δ* (ppm): 11.03 (s, 1H, indole NH), 10.03 (s, 1H, indole NH), 7.29–8.14 (m, 20H, Ar-H), 5.35 (s, 1H, –CH=), 2.44 (s, 3H, CH_3_); MS: *m/z* = 692 [M]^+∙^, 694 [M+2], 696 [M+4]; Anal. calcd. for (C_40_H_26_N_6_Cl_2_S): C, 69.26; H, 3.78; N, 12.12%. Found: C, 69.15; H, 3.69; N, 12.21%.

*3-(5-Chloro-3-phenyl-1H-indol-2-yl)-6-(4-chlorophenyl)-8-(5-methyl-2-phenyl-1H-indol-3-yl)-[1,2,4]triazolo[3,4-b][1,3,4]thiadiazepine (**3d**)*. IR (KBr) *ν*_max⁡_ (cm^−1^): 3391, 3265, 1601, 1540, 1519; ^1^H NMR (DMSO-d_6_+ CDCl_3_) *δ* (ppm): 11.43 (s, 1H, indole NH), 10.85 (s, 1H, indole NH), 6.40–9.13 (m, 20H, Ar-H), 4.91 (s, 1H, –CH=), 2.66 (s, 3H, CH_3_); MS: *m/z* = 692 [M]^+∙^, 694 [M+2], 696 [M+4]; Anal. calcd. for (C_40_H_26_N_6_Cl_2_S): C, 69.26; H, 3.78; N, 12.12%. Found: C, 69.15; H, 3.69; N, 12.21%.

*3-(5-Chloro-3-phenyl-1H-indol-2-yl)-8-(5-methyl-2-phenyl-1H-indol-3-yl)-6-phenyl-[1,2,4]triazolo[3,4-b][1,3,4]thiadiazepine (**3e**)*. IR (KBr) *ν*_max⁡_ (cm^−1^): 3106, 2996, 1650, 1590, 1560; ^1^H NMR (DMSO-d_6_+ CDCl_3_) *δ* (ppm): 11.01 (s, 1H, indole NH), 10.12 (s, 1H, indole NH), 6.40–8.58 (m, 21H, Ar-H), 4.91 (s, 1H, –CH=), 2.55 (s, 3H, CH_3_); MS: *m/z* = 658 [M]^+∙^, 660 [M+2]; Anal. calcd. for (C_40_H_27_N_6_ClS): C, 72.88; H, 4.13; N, 12.75%. Found: C, 72.75; H, 4.09; N, 12.64%.

*3-(5-Chloro-3-phenyl-1H-indol-2-yl)-8-(5-methyl-2-phenyl-1H-indol-3-yl)-6-(4-methylphenyl)-[1,2,4]triazolo[3,4-b][1,3,4]thiadiazepine (**3f**)*. IR (KBr) *ν*_max⁡_ (cm^−1^): 3443, 3133, 1602, 1578, 1558; ^1^H NMR (DMSO-d_6_+ CDCl_3_) *δ* (ppm): 11.31 (s, 1H, indole NH), 10.25 (s, 1H, indole NH), 7.11–8.18 (m, 20H, Ar-H), 5.29 (s, 1H, –CH=), 2.54 (s, 3H, CH_3_), 2.43 (s, 3H, CH_3_); MS: *m/z* = 672 [M]^+∙^, 674 [M+2]; Anal. calcd. for (C_41_H_29_N_6_ClS): C, 73.15; H, 4.34; N, 12.48%. Found: C, 73.02; H, 4.29; N, 12.37%.

*3-(5-Chloro-3-phenyl-1H-indol-2-yl)-6-(4-chlorophenyl)-8-(1H-indol-3-yl)-[1,2,4]triazolo[3,4-b][1,3,4]thiadiazepine (**3g**)*. IR (KBr) *ν*_max⁡_ (cm^−1^): 3239, 3098, 1607, 1578, 1553; ^1^H NMR (DMSO-d_6_+ CDCl_3_) *δ* (ppm): 11.78 (s, 1H, indole NH), 10.51 (s, 1H, indole NH), 6.40–8.56 (m, 17H, Ar-H), 4.94 (s, 1H, –CH=); MS: *m/z* = 602 [M]^+∙^, 604 [M+2], 606 [M+4]; Anal. calcd. for (C_33_H_20_N_6_Cl_2_S): C, 65.67; H, 3.34; N, 13.92; Found: C, 65.57; H, 3.28; N, 13.85%.

*3-(5-Chloro-3-phenyl-1H-indol-2-yl)-8-(1H-indol-3-yl)-6-phenyl-[1,2,4]triazolo[3,4-b][1,3,4]thiadiazepine (**3h**)*. IR (KBr) *ν*_max⁡_ (cm^−1^): 3404, 3104, 1608, 1558, 1505; ^1^H NMR (DMSO-d_6_+ CDCl_3_) *δ* (ppm): 10.61 (s, 1H, indole NH), 10.01 (s, 1H, indole NH), 6.43–8.91 (m, 18H, Ar-H), 5.15 (s, 1H, –CH=); MS: *m/z* = 568 [M]^+∙^, 570 [M+2]; Anal. calcd. for (C_33_H_21_N_6_ClS): C, 69.65; H, 3.72; N, 14.77%. Found: C, 69.55; H, 3.65; N, 14.71%.

*3-(5-Chloro-3-phenyl-1H-indol-2-yl)-8-(1H-indol-3-yl)-6-(4-methylphenyl)-[1,2,4]triazolo[3,4-b][1,3,4]thiadiazepine (**3i**)*. IR (KBr) *ν*_max⁡_ (cm^−1^): 3160, 3096, 1645, 1603; ^1^H NMR (DMSO-d_6_+ CDCl_3_) *δ* (ppm): 11.97 (s, 1H, indole NH), 11.39 (s, 1H, indole NH), 6.80–7.85 (m, 17H, Ar-H), 5.59 (s, 1H, –CH=), 2.64 (s, 3H, CH_3_); MS: *m/z* = 582 [M]^+∙^, 584 [M+2]; Anal. calcd. for (C_34_H_23_N_6_ClS): C, 70.03; H, 3.98; N, 14.41%. Found: C, 69.91; H, 3.95; N, 14.31%.

*3-(5-Bromo-3-phenyl-1H-indol-2-yl)-8-(5-chloro-2-phenyl-1H-indol-3-yl)-6-(4-chlorophenyl)-[1,2,4]triazolo[3,4-b][1,3,4]thiadiazepine (**3j**)*. IR (KBr) *ν*_max⁡_ (cm^−1^): 3148, 3098, 1643, 1589, 1551; ^1^H NMR (DMSO-d_6_+ CDCl_3_) *δ* (ppm): 12.48 (s, 1H, indole NH), 11.99 (s, 1H, indole NH), 7.07–8.23 (m, 20H, Ar-H), 5.60 (s, 1H, –CH=); MS: *m/z* = 756 [M]^+∙^, 758 [M+2], 760 [M+4], 762 [M+6]; Anal. calcd. for (C_39_H_23_N_6_BrCl_2_S): C, 61.75; H, 3.06; N, 11.08%. Found: C, 61.69; H, 3.01; N, 10.91%.

*3-(5-Bromo-3-phenyl-1H-indol-2-yl)-8-(5-chloro-2-phenyl-1H-indol-3-yl)-6-phenyl-[1,2,4]triazolo[3,4-b][1,3,4]thiadiazepine (**3k**)*. IR (KBr) *ν*_max⁡_ (cm^−1^): 3158, 3068, 1590, 1576, 1551; ^1^H NMR (DMSO-d_6_+ CDCl_3_) *δ* (ppm): 11.15 (s, 1H, indole NH), 10.05 (s, 1H, indole NH), 7.29–8.72 (m, 21H, Ar-H), 5.45 (s, 1H, –CH=); MS: *m/z* = 722 [M]^+∙^, 724 [M+2], 726 [M+4]; Anal. calcd. for (C_39_H_24_N_6_BrClS): C, 64.69; H, 3.34; N, 11.61%. Found: C, 65.21; H, 3.51; N, 11.45%.

*3-(5-Bromo-3-phenyl-1H-indol-2-yl)-8-(5-chloro-2-phenyl-1H-indol-3-yl)-6-(4-methylphenyl)-[1,2,4]triazolo[3,4-b][1,3,4]thiadiazepine (**3l**)*. IR (KBr) *ν*_max⁡_ (cm^−1^): 3108, 3029, 1644, 1606, 1553; ^1^H NMR (DMSO-d_6_+ CDCl_3_) *δ* (ppm): 11.03 (s, 1H, indole NH), 10.33 (s, 1H, indole NH), 7.2–8.1 (m, 20H, Ar-H), 5.05 (s, 1H, –CH=), 2.44 (s, 3H, CH_3_); MS: *m/z* = 736 [M]^+∙^, 738 [M+2], 740 [M+4]; Anal. calcd. for (C_40_H_26_N_6_BrClS): C, 65.09; H, 3.55; N, 11.39%. Found: C, 64.89; H, 3.51; N, 11.28%.

*3-(5-Bromo-3-phenyl-1H-indol-2-yl)-6-(4-chlorophenyl)-8-(5-methyl-2-phenyl-1H-indol-3-yl)-[1,2,4]triazolo[3,4-b][1,3,4]thiadiazepine (**3m**)*. IR (KBr) *ν*_max⁡_ (cm^−1^): 3176, 3048, 1623, 1584, 1509; ^1^H NMR (DMSO-d_6_+ CDCl_3_) *δ* (ppm): 12.20 (s, 1H, indole NH), 11.15 (s, 1H, indole NH), 6.32–8.13 (m, 20H, Ar-H), 5.60 (s, 1H, –CH=), 1.74 (s, 3H, CH_3_); MS: *m/z* = 736 [M]^+∙^, 738 [M+2], 740 [M+4]; Anal. calcd. for (C_40_H_26_N_6_BrClS): C, 65.09; H, 3.55; N, 11.39%. Found: C, 64.09; H, 3.51; N, 11.28%.

*3-(5-Bromo-3-phenyl-1H-indol-2-yl)-8-(5-methyl-2-phenyl-1H-indol-3-yl)-6-phenyl-[1,2,4]triazolo[3,4-b][1,3,4]thiadiazepine (**3n**)*. IR (KBr) *ν*_max⁡_ (cm^−1^): 3240, 3198, 1604, 1558, 1553; ^1^H NMR (DMSO-d_6_+ CDCl_3_) *δ* (ppm): 11.01 (s, 1H, indole NH), 9.90 (s, 1H, indole NH), 6.40–8.58 (m, 21H, Ar-H), 4.31 (s, 1H, –CH=), 2.55 (s, 3H, CH_3_); MS: *m/z* = 702 [M]^+∙^, 704 [M+2]; Anal. calcd. for (C_40_H_27_N_6_BrS): C, 68.28; H, 3.87; N, 11.94%. Found: C, 68.18; H, 3.82; N, 11.83%.

*3-(5-bromo-3-phenyl-1H-indol-2-yl)-8-(5-methyl-2-phenyl-1H-indol-3-yl)-6-(4-methylphenyl)-[1,2,4]triazolo[3,4-b][1,3,4]thiadiazepine (**3o**)*. IR (KBr) *ν*_max⁡_ (cm^−1^): 3117, 3047, 1641, 1606, 1573; ^1^H NMR (DMSO-d_6_+ CDCl_3_) *δ* (ppm): 10.25 (s, 1H, indole NH), 9.95 (s, 1H, indole NH), 7.11–8.18 (m, 20H, Ar-H), 5.15 (s, 1H, –CH=), 2.54 (s, 3H, CH_3_), 2.43 (s, 3H, CH_3_); MS: *m/z* = 716 [M]^+∙^, 718 [M+2]; Anal. calcd. for (C_41_H_29_N_6_BrS): C, 68.62; H, 4.07; N, 11.71%. Found: C, 68.52; H, 4.05; N, 11.59%.

*3-(5-Bromo-3-phenyl-1H-indol-2-yl)-6-(4-chlorophenyl)-8-(1H-indol-3-yl)-[1,2,4]triazolo[3,4-b][1,3,4]thiadiazepine (**3p**)*. IR (KBr) *ν*_max⁡_ (cm^−1^): 3167, 3047, 1648, 1589, 1558; ^1^H NMR (DMSO-d_6_+ CDCl_3_) *δ* (ppm): 10.61 (s, 1H, indole NH), 10.23 (s, 1H, indole NH), 6.83–8.19 (m, 17H, Ar-H), 5.19 (s, 1H, –CH=); MS: *m/z* = 646 [M]^+∙^, 648 [M+2], 650 [M+4]; Anal. calcd. for (C_33_H_20_N_6_BrClS): C, 61.17; H, 3.11; N, 12.97%. Found: C, 61.12; H, 3.09; N, 12.85%.

*3-(5-Bromo-3-phenyl-1H-indol-2-yl)-8-(1H-indol-3-yl)-6-phenyl-[1,2,4]triazolo[3,4-b][1,3,4]thiadiazepine (**3q**)*. IR (KBr) *ν*_max⁡_ (cm^−1^): 3097, 2998, 1606, 1578, 1551; ^1^H NMR (DMSO-d_6_+ CDCl_3_) *δ* (ppm): 11.01 (s, 1H, indole NH), 10.01 (s, 1H, indole NH), 6.83–8.91 (m, 18H, Ar-H), 5.15 (s, 1H, –CH=); MS: *m/z* = 612 [M]^+∙^, 614 [M+2]; Anal. calcd. For (C_33_H_21_N_6_BrS): C, 64.60; H, 3.45; N, 13.70%. Found: C, 64.56; H, 3.41; N, 13.51%.

*3-(5-Bromo-3-phenyl-1H-indol-2-yl)-8-(1H-indol-3-yl)-6-(4-methylphenyl)-[1,2,4]triazolo[3,4-b][1,3,4]thiadiazepine (**3r**)*. IR (KBr) *ν*_max⁡_ (cm^−1^): 3104, 3049, 1608, 1598, 1558; ^1^H NMR (DMSO-d_6_+ CDCl_3_) *δ* (ppm): 11.07 (s, 1H, indole NH), 10.19 (s, 1H, indole NH), 6.80–7.85 (m, 17H, Ar-H), 5.39 (s, 1H, –CH=) 2.64 (s, 3H, CH_3_); MS: *m/z* = 626 [M]^+∙^, 628 [M+2]; Anal. calcd. for (C_34_H_23_N_6_BrS): C, 65.07; H, 3.69; N, 13.39%. Found: C, 64.95; H, 3.65; N, 13.28%.

*8-(5-Chloro-2-phenyl-1H-indol-3-yl)-6-(4-chlorophenyl)-3-(5-methyl-3-phenyl-1H-indol-2-yl)-[1,2,4]triazolo[3,4-b][1,3,4]thiadiazepine (**3s**)*. IR (KBr) *ν*_max⁡_ (cm^−1^): 3219, 3196, 1641, 1589, 1552; ^1^H NMR (DMSO-d_6_+ CDCl_3_) *δ* (ppm): 11.01 (s, 1H, indole NH), 10.25 (s, 1H, indole NH), 6.40–8.59 (m, 20H, Ar-H), 4.95 (s, 1H, –CH=), 2.56 (s, 3H, CH_3_); MS: *m/z* = 692 [M]^+∙^, 694 [M+2], 696 [M+4]; Anal. calcd. for (C_40_H_26_N_6_Cl_2_S): C, 69.26; H, 3.78; N, 12.12%. Found: C, 69.14; H, 3.72; N, 12.02%.

*8-(5-Chloro-2-phenyl-1H-indol-3-yl)-3-(5-methyl-3-phenyl-1H-indol-2-yl)-6-phenyl-[1,2,4]triazolo[3,4-b][1,3,4]thiadiazepine (**3t**).* IR (KBr) *ν*_max⁡_ (cm^−1^): 3244, 3189, 1641, 1604, 1552; ^1^H NMR (DMSO-d_6_+ CDCl_3_) *δ* (ppm): 12.39 (s, 1H, indole NH), 11.09 (s, 1H, indole NH), 6.80–7.85 (m, 21H, Ar-H), 5.15 (s, 1H, –CH=), 2.76 (s, 3H, CH_3_); MS: *m/z* = 658 [M]^+∙^, 660 [M+2]; Anal. calcd. for (C_40_H_27_N_6_ClS): C, 72.88; H, 4.13; N, 12.75%. Found: C, 72.78; H, 4.10; N, 12.59%.

*8-(5-Chloro-2-phenyl-1H-indol-3-yl)-3-(5-methyl-3-phenyl-1H-indol-2-yl)-6-(4-methylphenyl)-[1,2,4]triazolo[3,4-b][1,3,4]thiadiazepine (**3u**)*. IR (KBr) *ν*_max⁡_ (cm^−1^): 3248, 3198, 1606, 1579, 1552; ^1^H NMR (DMSO-d_6_+ CDCl_3_) *δ* (ppm): 12.20 (s, 1H, indole NH), 11.98 (s, 1H, indole NH), 7.05–8.13 (m, 20H, Ar-H), 4.37 (s, 1H, –CH=), 2.57 (s, 3H, CH_3_), 2.01 (s, 3H, CH_3_); MS: *m/z* = 672 [M]^+∙^, 674 [M+2]; Anal. calcd. for (C_41_H_29_N_6_ClS): C, 73.15; H, 4.34; N, 12.48%. Found: C, 73.28; H, 4.31; N, 12.36%.

*6-(4-Chlorophenyl)-8-(5-methyl-2-phenyl-1H-indol-3-yl)-3-(5-methyl-3-phenyl-1H-indol-2-yl)-[1,2,4]triazolo[3,4-b][1,3,4]thiadiazepine (**3v**)*. IR (KBr) *ν*_max⁡_ (cm^−1^): 3248, 3198, 1604, 1574, 1552; ^1^H NMR (DMSO-d_6_+ CDCl_3_) *δ* (ppm): 11.39 (s, 1H, indole NH), 10.39 (s, 1H, indole NH), 6.43–8.91 (m, 20H, Ar-H), 4.55 (s, 1H, –CH=), 2.58 (s, 3H, CH_3_); MS: *m/z* = 672 [M]^+∙^, 674 [M+2]; Anal. calcd. for (C_41_H_29_N_6_ClS): C, 73.15; H, 4.34; N, 12.48%. Found: C, 73.28; H, 4.31; N, 12.36%.

*8-(5-Methyl-2-phenyl-1H-indol-3-yl)-3-(5-methyl-3-phenyl-1H-indol-2-yl)-6-phenyl-[1,2,4]triazolo[3,4-b][1,3,4]thiadiazepine (**3w**)*. IR (KBr) *ν*_max⁡_ (cm^−1^): 3248, 3179, 1604, 1574, 1556; ^1^H NMR (DMSO-d_6_+ CDCl_3_) *δ* (ppm): 11.05 (s, 1H, indole NH), 10.10 (s, 1H, indole NH), 6.32–8.13 (m, 21H, Ar-H), 5.60 (s, 1H, –CH=), 2.23 (s, 6H, CH_3_); MS: *m/z* = 638 [M]^+∙^; Anal. calcd. For (C_41_H_30_N_6_S): C, 77.09; H, 4.73; N, 13.16%. Found: C, 77.06; H, 4.68; N, 13.03%.

*8-(5-Methyl-2-phenyl-1H-indol-3-yl)-3-(5-methyl-3-phenyl-1H-indol-2-yl)-6-(4-methylphenyl)-[1,2,4]triazolo[3,4-b][1,3,4]thiadiazepine (**3x**)*. IR (KBr) *ν*_max⁡_ (cm^−1^): 3184, 3148, 1606, 1574, 1553; ^1^H NMR (DMSO-d_6_+ CDCl_3_) *δ* (ppm): 11.05 (s, 1H, indole NH), 10.07 (s, 1H, indole NH), 6.40–8.77 (m, 20H, Ar-H), 4.15 (s, 1H, –CH=), 2.54 (s, 6H, CH_3_), 2.31 (s, 3H, CH_3_); MS: *m/z* = 652 [M]^+∙^; Anal. calcd. for (C_42_H_32_N_6_S): C, 77.27; H, 4.94; N, 12.87%. Found: C, 77.17; H, 4.91; N, 12.96%.

*8-(1H-Indol-3-yl)-3-(5-methyl-3-phenyl-1H-indol-2-yl)-6-phenyl-[1,2,4]triazolo[3,4-b][1,3,4]thiadiazepine (**3y**)*. IR (KBr) *ν*_max⁡_ (cm^−1^): 3354, 3258, 1674, 1595, 1554; ^1^H NMR (DMSO-d_6_+ CDCl_3_) *δ* (ppm): 10.61 (s, 1H, indole NH), 10.01 (s, 1H, indole NH), 6.40–8.91 (m, 18H, Ar-H), 4.85 (s, 1H, –CH=), 2.54 (s, 3H, CH_3_); MS: *m/z* = 548 [M]^+∙^; Anal. calcd. for (C_34_H_24_N_6_S): C, 74.43; H, 4.41; N, 15.32%. Found: C, 74.39; H, 4.39; N, 15.25%.

*6-(4-Chlorophenyl)-8-(1H-indol-3-yl)-3-(5-methyl-3-phenyl-1H-indol-2-yl)-[1,2,4]triazolo[3,4-b][1,3,4]thiadiazepine (**3z**)*. IR (KBr) *ν*_max⁡_ (cm^−1^): 3391, 3244, 1667, 1601, 1540; ^1^H NMR (DMSO-d_6_+ CDCl_3_) *δ* (ppm): 12.23 (s, 1H, indole NH), 10.11 (s, 1H, indole NH), 6.76–7.61 (m, 17H, Ar-H), 4.37 (s, 1H, –CH=), 2.08 (s, 3H, CH_3_); MS: *m/z* = 582 [M]^+∙^ 584 [M+2]; Anal. calcd. For (C_34_H_25_N_6_S): C, 70.03; H, 3.98; N, 14.41%. Found: C, 69.98; H, 3.95; N, 14.35%.

### 2.2. Biological Activities

#### 2.2.1. Antioxidant Activities

*(1) Free Radical Scavenging Activity.* Free radical scavenging activity was done by DPPH method [32]. Different concentrations (25 *μ*g, 50 *μ*g, and 100 *μ*g) of samples and butylated hydroxy anisole (BHA) were taken in different test tubes. The volume was adjusted to 100 *μ*L by adding MeOH. Five milliliters of 0.1 mM methanolic solution of DPPH was added to these tubes and shaken vigorously. The tubes were allowed to stand at 27°C for 20 min. The control was prepared as above without any samples. The absorbances of samples were measured at 517 nm. Radical scavenging activity was calculated using the following formula:

(1) %  Radical  scavenging  activity =[(Control  OD−Sample  OD)(Control  OD)]×100.

*(2) Total Antioxidant Capacity.* Various concentrations of samples (25 *μ*g, 50 *μ*g, and 100 *μ*g) were taken in a series of test tubes. To this, 1.9 mL of reagent solution (0.6 M sulfuric acid, 28 mM sodium phosphate, and 4 mM ammonium molybdate) was added. The tubes were incubated at 95°C for 90 min and allowed to cool. The absorbance of each aqueous solution was measured at 695 nm against a blank. Antioxidant capacities are expressed as equivalents of ascorbic acid. Ascorbic acid equivalents were calculated using standard graph of ascorbic acid. The values are expressed as ascorbic acid equivalents in *μ*g per mg of samples.

*(3) Ferric Reducing Antioxidant Power.* Various concentrations of samples (25 *μ*g, 50 *μ*g, and 100 *μ*g) were mixed with 2.5 mL of 200 mmol/L sodium phosphate buffer (pH 6.6) and 2.5 mL of 1% potassium ferricyanide. The mixture was incubated at 50°C for 20 min. Next, 2.5 mL of 10% trichloroacetic acid (w/v) was added. From this solution, 5 mL was mixed with 5 mL of distilled water and 1 mL of 0.1% ferric chloride and absorbance was measured spectrophotometrically at 700 nm. BHA was used as standard.

### 2.3. Antimicrobial Activity

Series of novel indole analogues are tested for *in vitro* antimicrobial activity against gram-negative bacteria *Escherichia coli* ATCC 25922 *and Klebsiella pneumoniae* ATCC 33499 and gram-positive bacteria *Staphylococcus aureus* ATCC 6538 and antifungal activity against *Candida tropicalis* ATCC 8302 and *Candida albicans* ATCC 60193by applying the agar plate diffusion technique [33]. Dilution process was adopted at 25 *μ*g, 50 *μ*g, and 100 *μ*g/mL concentrations, respectively. The activity is compared with reference drugs gentamycin for antibacterial and fluconazole for antifungal activity. The zone of inhibition after 24 hr of incubation at 37°C in case of antibacterial activity and 48 hr in case of antifungal activity was compared with that of standards.

## 3. Results and Discussion

### 3.1. Chemistry

**Scheme 1:**
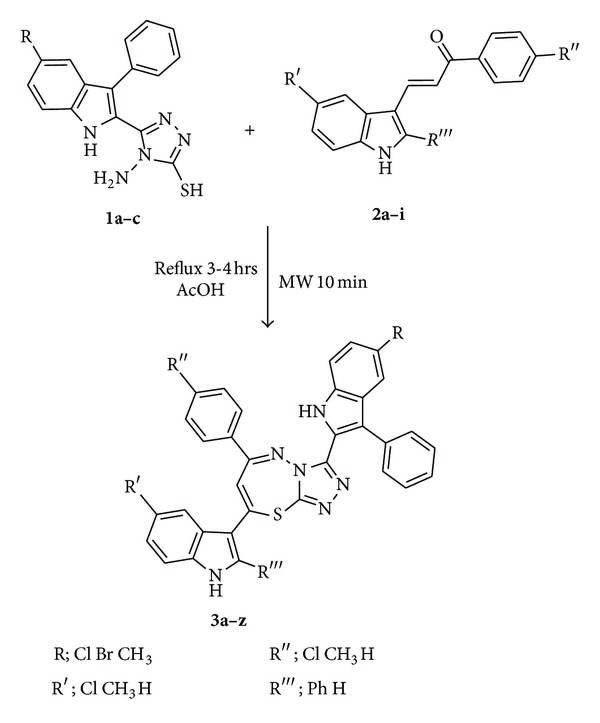
Schematic representation for the formation of novel triazolothiadiazepinylindole **3a**–**z**.

Molecules were designed with the aim of exploring their antioxidant and antimicrobial activities. The target compounds were synthesized as outlined in (Scheme 1). 3,5-Disubstitutedindole-2-carboxyhydrazides were reacted with carbon disulphide in the presence of base and hydrazine hydrate to get 5-(5-substituted-3-phenyl-1H-indol-2-yl)-4-amino-4H-1,2,4-triazole-3-thiols **1a**–**c**. Claisen-Schmidt condensation of 2,5-disubstituted indole-3-carboxaldehydes with substituted acetophenones produced 3-(2,5-disubstituted-1H-indol-3-yl)-1-(4-substituted-phenyl)prop-2-en-1-one **2a**–**i**. The synthesized compounds **3a**–**z** were obtained in good yield by cyclocondensation of 5-(5-substituted-3-phenyl-1H-indol-2-yl)-4-amino-4H-1,2,4-triazole-3-thiol **1a**–**c** with 3-(2,5-disubstituted-1H-indol-3-yl)-1(4-substituted phenyl)prop-2-en-1-one **2a**–**i**. The formation of products was monitored by TLC. All the newly synthesized compounds were characterized by IR, ^1^H NMR, ^13^C NMR, mass spectroscopic and analytical data. The IR spectrum of 8-(5-chloro-2-phenyl-1H-indol-3-yl)-3-(5-chloro-3-phenyl-1H-indol-2-yl)-6-(3-chlorophenyl)-[1,2,4]triazolo[3,4-b][1,3,4]thiadiazepine **3a** showed a strong absorption at 3180 cm^−1^ and 3090 cm^−1^ corresponding to indole NH, absorption at 1654 and 1624, corresponding to triazole C=N, and absorption at 1546 cm^−1^ corresponding to thiadiazepine C=N stretching, respectively. The ^1^H NMR spectrum of **3a** has exhibited a singlet at *δ* 12.47 ppm due to indole NH and peak at *δ* 11.63 ppm is due to indole NH which is also D_2_O exchangeable. A multiplet between *δ* 7.31–8.47 ppm corresponds to twenty aromatic protons present in the molecule and a peak at *δ* 5.65 ppm is assigned for the –CH= of thiadiazepine ring proton. The ^13^C NMR spectrum of compound **3a** has shown peaks at *δ* 108, 111, 113, 117, 118, 118, 118, 120, 123, 125, 126, 126, 126, 128, 128, 128, 128, 129, 129, 129, 130, 132, 133, 134, 135, 138, 138, 144, 145, and 166. The mass spectrum of compound **3a** has shown molecular ion peak at *m/z* 712 [M]^+∙^ which is corresponding to molecular weight of the compound. The above spectral data supports the formation of compound **3a**.

Various new triazolothiadiazepinylindole analogues synthesized during the present investigation are listed in (Table 1).

### 3.2. Biological Activities

The compounds **3a**–**z** were screened for their antioxidant (free radical scavenging, total antioxidant capacity, and ferric reducing antioxidant power) and antimicrobial activities.

#### 3.2.1. Antioxidant Activities

**Figure 1:**
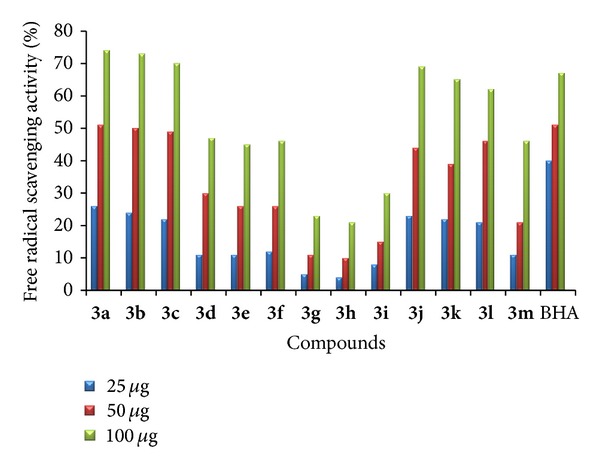
Free radical scavenging activity of **3a**–**m**.

**Figure 2:**
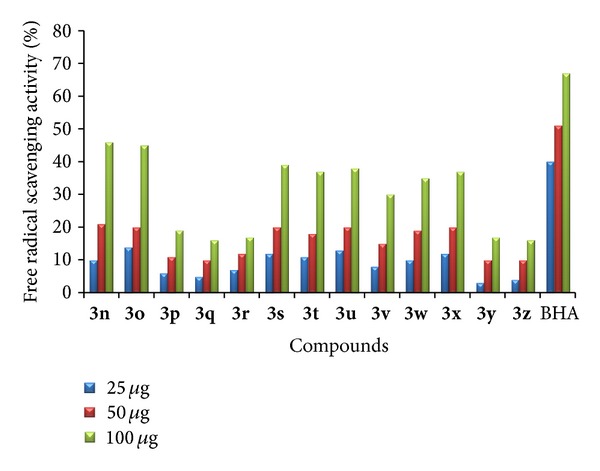
Free radical scavenging activity of **3n**–**z**.

*(1) Free Radical Scavenging Activity.* The target compounds were screened for free radical scavenging activity by DPPH method [32]. The samples were prepared at concentrations of 25, 50, and 100 *μ*g/100 *μ*L and butylated hydroxy anisole (BHA) was taken as standard. DPPH is a stable free radical in a methanolic solution. Because of the unpaired electron of DPPH, it gives a strong absorption maxima at 517 nm in the visible region (purple color). In addition, the unpaired electron of the radical becomes paired in the presence of a hydrogen donor (a free radical scavenging antioxidant), decreasing the absorption. Among the compounds tested **3a**–**c** and **3j**–**l** have shown very promising free radical scavenging activity. The increased activity is due to the existence of halogen substitution at the five positions of both indoles. The hydrogen of indole NH could be donated to the DPPH to form DPPH free radical; by the presence of phenyl ring at the third position of indole, the DPPH free radical will be stabilized by the resonance. Compounds **3d**–**f**, **3m**–**o,** and **3s**–**x** containing halogen atom at five positions of indole and a methyl group at another indole ring have shown moderate activity, whereas compounds **3g**–**i**, **3p**–**r,** and **3y**–**z** have shown the least activity compared with the standard. The bar graph representation of percentage of free radical scavenging activity is displayed in Figures 1 and 2.

**Figure 3:**
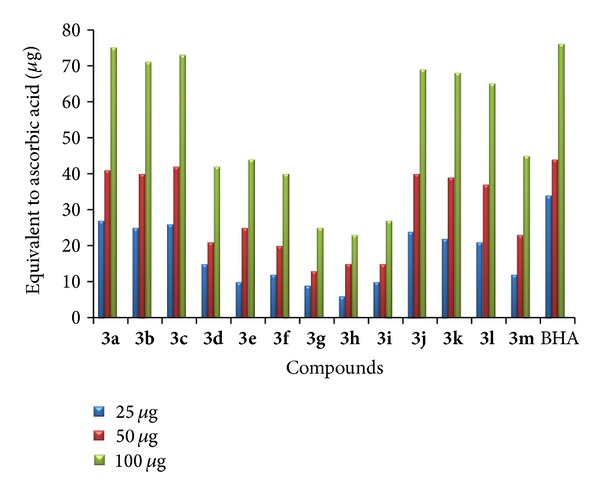
Total antioxidant capacity of **3a**–**m**.

**Figure 4:**
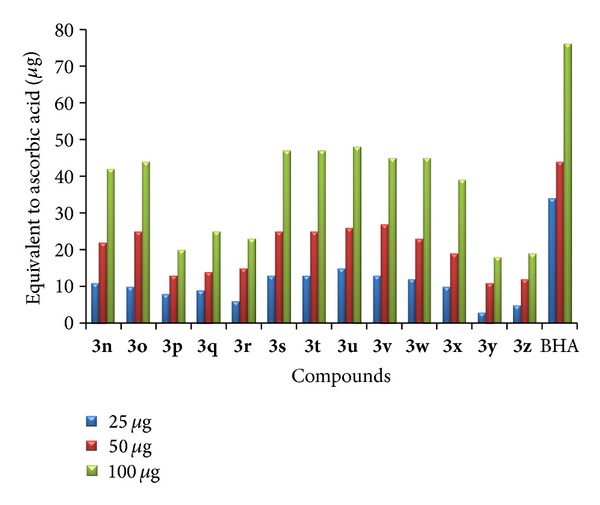
Total antioxidant capacity of **3n**–**z**.

*(2) Total Antioxidant Capacity.* Total antioxidant activity was performed to all the newly synthesized compounds [34]. Antioxidant capacities are expressed as equivalents of ascorbic acid. Among the tested compounds **3a**–**c** and **3j**–**l** which are halogen substituted triazolothiadiazepinylindole have shown very strong total antioxidant capacity. Compounds with methyl substitution at the fifth position of the indole ring and no substitution at the second and fifth positions have shown the least total antioxidant capacity compared with the standard. The increased activity is due to the presence of halogen at the fifth position and a phenyl ring at the third position of indole. The results of total antioxidant activity are shown in Figures 3 and 4.

**Figure 5:**
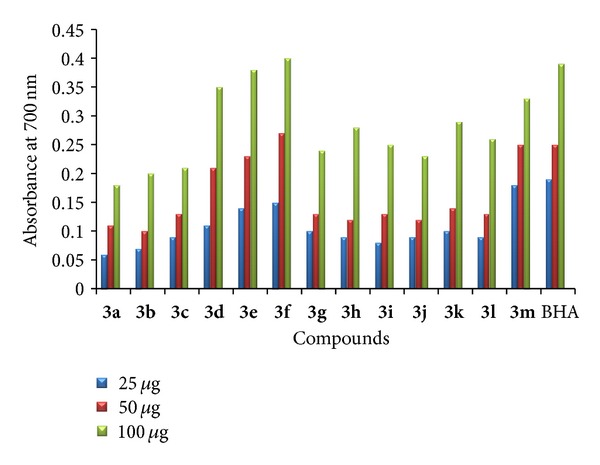
Ferric reducing antioxidant power activity of **3a**–**m**.

**Figure 6:**
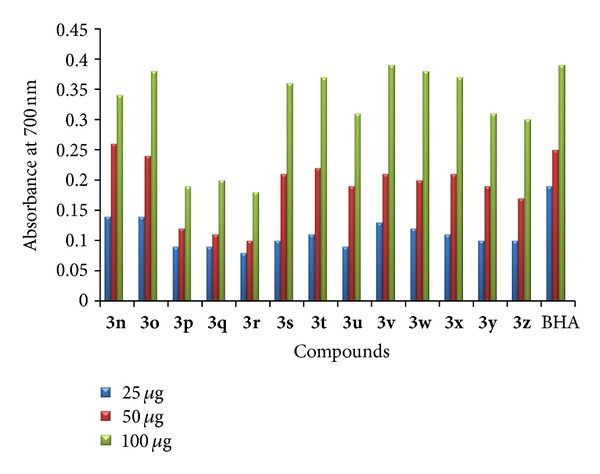
Ferric reducing antioxidant power activity of **3n**–**z**.

*(3) Ferric Reducing Antioxidant Power Activity*. The novel compounds were screened for ferric reducing antioxidant activity [35]. Butylated hydroxy anisole (BHA) was used as standard. All the tested compounds have shown positive tendency towards the ferric reducing activity. The presence of reducer (i.e., antioxidant) causes the reduction of the Fe^+3^/ferricyanide complex to the Fe^+2^ form after the addition of trichloroacetic acid and ferric chloride. The reducing power of test compounds increases with increase in concentration. Compounds **3d**–**f**, **3m**–**o,** and **3s**–**z** have shown excellent ferric reducing antioxidant activity and other analogues of indole have shown moderate to high activity. The presence of methyl group at the fifth position of the indole ring plays an important role as a better electron donor which enhances reducing power activity of the compounds. The results are presented in Figures 5 and 6.

### 3.3. Antimicrobial Activity

Applying the agar plate diffusion technique [33], series of novel triazolothiadiazepinylindole analogues were screened for *in vitro* antibacterial activity against (Table 2) gram-negative bacteria *Escherichia coli* (*E. coli*)* and Klebsiella pneumoniae *(*K. pneumoniae*) and gram-positive bacteria *Staphylococcus aureus *(*S. aureus*) at 25 *μ*g/mL, 50 *μ*g/mL, and 100 *μ*g/mL concentrations, respectively. Gentamycin was used as standard. The zone of inhibitions was measured in mm for each concentration. Most of the screened compounds were found to have significant antibacterial activity. Compounds **3a**–**c** and **3j**–**l** have shown very good activity against all the three bacterial strains. Compounds **3d**–**f**, **3m**–**o,** and **3s**–**x** have shown moderate activity and compounds **3g**–**i**, **3p**–**r,** and **3y**–**z** have shown the least activity. Antifungal screening of the compounds was carried out *in vitro* against two fungi strains *Candida tropicalis* and *Candida albicans* at 25 *μ*g/mL, 50 *μ*g/mL, and 100 *μ*g/mL concentrations using fluconazole as standard. Among the tested indole analogues the majority of compounds exhibited moderate to significant antifungal activity.

## 4. Conclusions

We have synthesized titled compounds **3a**–**z** by economic, better yield, and safer methods through the formation of compounds **1a**–**c** and **2a**–**i** under thermal and microwave condition. The compounds **3a**–**z** were subjected for their antioxidant and antimicrobial screening. Very potent antimicrobial, scavenging and antioxidant activity was observed with compounds containing halogens at the fifth position of indoles. Excellent ferric reducing activity was observed with compounds containing electron donor group at five positions of one/both indoles. Therefore, the findings will provide a great impact on chemists and biochemists for further investigations in the indole field in search of molecules possessing potent antioxidant and antimicrobial activities.

## Figures and Tables

**Table 1:** Comparative data of conventional and microwave methods for the synthesis of novel triazolothiadiazepinylindole **3a–z**.

Compd^a^ Number	Substituents				Conventional method		Microwave method		m.p.^c^ (°C)
	R	R′	R′′	R′′′	Time (min)	Yield^b^ (%)	Time (min)	Yield^b^ (%)	
**3a**	Cl	Cl	Cl	Ph	180–240	85	10	95	200–02
**3b**	Cl	Cl	H	Ph	180–240	80	10	93	142–43
**3c**	Cl	Cl	Me	Ph	180–240	75	10	95	194–96
**3d**	Cl	Me	Cl	Ph	180–240	80	10	95	160–62
**3e**	Cl	Me	H	Ph	180–240	70	10	93	190–92
**3f**	Cl	Me	Me	Ph	180–240	65	10	95	158–60
**3g**	Cl	H	Cl	H	180–240	60	10	85	195–97
**3h**	Cl	H	H	H	180–240	60	10	80	168–70
**3i**	Cl	H	Me	H	180–240	70	10	90	155–57
**3j**	Br	Cl	Cl	Ph	180–240	80	10	98	210–12
**3k**	Br	Cl	H	Ph	180–240	85	10	96	195–97
**3l**	Br	Cl	Me	Ph	180–240	85	10	95	140–42
**3m**	Br	Me	Cl	Ph	180–240	75	10	90	180–82
**3n**	Br	Me	H	Ph	180–240	65	10	85	165–67
**3o**	Br	Me	Me	Ph	180–240	60	10	80	168–70
**3p**	Br	H	Cl	H	180–240	60	10	85	210–12
**3q**	Br	H	H	H	180–240	60	10	75	218–20
**3r**	Br	H	Me	H	180–240	60	10	80	120–22
**3s**	Me	Cl	Cl	Ph	180–240	75	10	85	183–85
**3t**	Me	Cl	H	Ph	180–240	75	10	85	201–02
**3u**	Me	Cl	Me	Ph	180–240	80	10	87	181–83
**3v**	Me	Me	Cl	Ph	180–240	65	10	85	190–92
**3w**	Me	Me	H	Ph	180–240	60	10	80	161–62
**3x**	Me	Me	Me	Ph	180–240	65	10	86	172–74
**3y**	Me	H	H	H	180–240	60	10	75	158–60
**3z**	Me	H	Cl	H	180–240	60	10	70	149–51

^a^Products were characterized by IR, ^1^H NMR,^13^C NMR, MS, and elemental analysis. ^b^Isolated yield. ^c^Melting points are uncorrected.

**Table 2:** Zone of inhibition in mm at 25, 50, and 100 µg/mL concentrations.

Compd name	Antibacterial activity									Antifungal activity					
	*S. aureus *			*E. coli *			*K. pneumoniae *			*C. tropicalis *			*C. albicans *		
	25	50	100	25	50	100	25	50	100	25	50	100	25	50	100
**3a**	**13**	**17**	**20**	**15**	**20**	**22**	**16**	**22**	**25**	**14**	**18**	**20**	**13**	**15**	**18**
**3b**	**14**	**16**	**20**	**16**	**19**	**24**	**15**	**21**	**24**	**15**	**17**	**21**	**14**	**17**	**19**
**3c**	**15**	**15**	**19**	**14**	**21**	**25**	**14**	**23**	**26**	**13**	**16**	**20**	**15**	**16**	**20**
**3d**	11	12	17	12	15	16	09	14	15	10	12	15	10	12	14
**3e**	10	13	15	11	16	17	08	15	18	09	11	14	09	11	12
**3f**	09	12	16	09	14	16	09	13	14	09	12	13	09	10	10
**3g**	02	06	08	05	07	08	05	07	09	02	04	06	03	04	05
**3h**	03	04	09	03	05	07	04	08	10	01	03	05	03	06	09
**3i**	05	07	08	04	06	08	03	06	08	03	04	06	05	06	08
**3j**	**14**	**18**	**21**	**18**	**18**	**23**	**15**	**21**	**23**	**15**	**18**	**21**	**13**	**15**	**17**
**3k**	**13**	**19**	**20**	**17**	**20**	**22**	**17**	**23**	**24**	**16**	**19**	**21**	**15**	**17**	**18**
**3l**	**12**	**18**	**21**	**16**	**19**	**25**	**16**	**22**	**26**	**12**	**15**	**21**	**13**	**16**	**19**
**3m**	10	10	15	10	12	15	09	15	16	10	12	15	09	11	12
**3n**	08	11	14	08	13	14	10	13	18	09	11	14	09	09	11
**3o**	08	10	14	09	14	16	09	13	19	08	10	12	10	10	12
**3p**	03	04	09	04	05	08	06	08	12	04	06	08	03	05	07
**3q**	03	05	07	03	05	08	05	09	11	03	05	07	05	06	08
**3r**	04	06	08	06	08	09	04	08	10	02	05	06	03	06	07
**3s**	10	10	15	10	12	15	09	14	15	10	12	14	11	12	13
**3t**	09	11	13	11	14	17	10	15	18	08	11	15	09	10	12
**3u**	08	11	14	08	13	14	10	13	14	09	13	13	08	12	14
**3v**	09	09	16	10	12	15	11	12	17	10	12	15	09	13	15
**3w**	08	10	14	09	14	16	09	12	15	09	11	16	10	11	12
**3x**	09	12	12	09	11	14	08	14	16	08	10	17	09	10	12
**3y**	04	06	09	05	08	10	05	09	10	05	09	10	04	05	06
**3z**	05	05	08	04	09	11	04	10	12	04	08	11	04	04	05

**Std.1**	15	19	22	18	21	25	17	23	27	—	—	—	—	—	—
**Std.2**	—	—	—	—	—	—	—	—	—	15	19	22	16	19	21

Std.1: gentamycin, Std.2: fluconazole.

The bold font refers to the compounds which have shown more potent antimicrobial activities.
